# Nutraceuticals for Dyslipidaemia and Glucometabolic Diseases: What the Guidelines Tell Us (and Do Not Tell, Yet)

**DOI:** 10.3390/nu14030606

**Published:** 2022-01-30

**Authors:** Manuela Casula, Alberico Luigi Catapano, Paolo Magni

**Affiliations:** 1 Dipartimento di Scienze Farmacologiche e Biomolecolari, Università degli Studi di Milano, 20133 Milan, Italy; manuela.casula@unimi.it (M.C.); alberico.catapano@unimi.it (A.L.C.); 2 IRCCS MultiMedica, Sesto San Giovanni, 20099 Milan, Italy

**Keywords:** guidelines, nutraceutical product, cardiovascular disease, dyslipidaemia, metabolic disease, type 2 diabetes mellitus

## Abstract

Background: The use of nutraceutical products and functional foods in the cardiovascular and metabolic field is rising in several countries. Preparation and implementation of guidelines are pivotal for translating research-derived knowledge and evidence-based medicine to the clinical practice. Based on these considerations, the aim of this paper is to explore if and how nutraceutical products are discussed by the most recent international guidelines related to cardio-metabolic diseases (dyslipidaemia, obesity, type 2 diabetes mellitus (T2DM) and cardiovascular disease (CVD) prevention). Some, but not all, guidelines for dyslipidaemia mention nutraceutical products as potential useful options for the treatment of mild dyslipidaemia, but also indicate the low level of evidence associated to their effects on hard endpoints (myocardial infarction, stroke, CVD-related death). In the most recent guidelines on obesity, it is mentioned that no safe and effective dietary supplement nor nutraceutical product is available for the management of weight loss in this condition, and more high-quality studies are necessary in this field. The examined guidelines for T2DM do not mention any specific nutraceutical approach to this disease, nor to milder forms, such as insulin resistance and pre-diabetes. Conclusions: The focus on nutraceutical products in the main international guidelines for cardio-metabolic disease management remains limited. Since robust scientific evidence is the background of useful and effective guidelines, the implementation of high-quality clinical research is strongly needed in the field of nutraceutical products for cardio-metabolic diseases.

## 1. Introduction

Cardiovascular (CV) and metabolic diseases are the leading cause of morbidity, disability and mortality in the world [1] and their heterogenous causes are currently on the rise [2]. Their epidemiological and economic impact, thus, poses a significant challenge and requires a multifactorial approach, starting from the promotion of a healthy lifestyle and, when necessary, the use of preventive or treatment therapies, along with appropriate first-level and second-level diagnostic procedures. Indeed, over time, multiple diagnostic and treatment options have become available, including increasingly effective and safer medications, which also allow a more personalised approach. The current clinical practice in many fields and, in particular, in the CV disease (CVD) and metabolic disease area, is built on a body of knowledge and evidence, which is collected in guidelines focusing on specific medical issues [3,4]. These guidelines are constructed according to a specific development process [5,6], which takes into consideration several aspects, including a systematic review of the available evidence, the inclusion of a multidisciplinary panel of representatives and experts from key affected groups (such as patients), and the identification of potential conflicts of interest. Most important, they should periodically undergo reconsideration and revision, as appropriate according to any important novel evidence that arises. Among the treatment options, the use of nutraceutical products is currently growing in several biomedical areas, including the cardiometabolic one and, thus, requires a robust scientific validation. Nutraceuticals are nutritional compounds of natural origin, shown to be efficacious in preventive medicine or in the treatment or co-treatment of diseases. Specific to this review, several foods and dietary supplements have been shown to contribute to protection against the development of CVD [7]. However, within the landscape of intervention options, the role of nutraceutical products and functional foods, especially in the cardio-metabolic field, is still rather limited in the area of prescription medications, albeit variably depending on geographic context and disease type [8]. In any case, the overall nutraceutical market appears to have quite large proportions worldwide and the specific field of nutraceuticals for CVD and metabolic disease prevention may be expected to grow in the next years, as well. Thus, the discussion about the usefulness of nutraceutical options and the related potential critical issues in this area begin to be mentioned in the guidelines related to the metabolic field.

Based on these considerations, in this review, we aim at exploring if and how nutraceutical products are considered and discussed by the most recent guidelines devoted to the management of CV and metabolic diseases, with specific regards to dyslipidaemia, a major causative factor for atherosclerotic CVD (ASCVD), as well as obesity and type 2 diabetes mellitus (T2DM). This review is based upon the analysis of the international guidelines regarding CVDs, obesity, T2DM and metabolic syndrome, published in the last 20 years, in order to assess the relevance of this topic over time and in different regions of the world.

## 2. Nutraceutical Products and Guidelines for Dyslipidaemia

The use of nutraceutical products for the management of dyslipidaemia (and some features, such as insulin resistance, which are often associated to it) is a rather diffused option and the efficacy and safety of several such products, mostly combining two or more active phytoextracts or compounds, have been evaluated by good-quality RCTs [9,10,11,12,13].

Taking into consideration specific guidelines (Table 1), in the last version of the 2019 European Society of Cardiology (ESC)/European Society of Atherosclerosis (EAS) Guidelines for the management of dyslipidaemias [4], authors stressed the central role of nutrition for the prevention of ASCVD, but also the weakness of evidence and the lack of concordance among studies, suggesting caution in interpreting the results of randomised controlled trials (RCTs). These guidelines list a number of dietary supplements and functional foods to be considered for the treatment of dyslipidaemias, which are briefly discussed below. Plant sterols and stanols are phytosterols, and have been identified in several plant products, including various fruits and vegetables, cereals, nuts and seeds. Their biological activity is derived from their molecular structural similarity to cholesterol. According to the guidelines, in compliance with the LDL-C goal and without any safety contraindications, plant sterols/stanols “may be considered: (i) in individuals with high cholesterol levels at intermediate or low global CV risk who do not qualify for pharmacotherapy; (ii) as an adjunct to pharmacological therapy in high- and very-high-risk patients who fail to achieve LDL-C goals on statins or could not be treated with statins; and (iii) in adults and children (aged >6 years) with familial hypercholesterolemia (FH)” [4]. The role of phytosterols in controlling cholesterol levels according to the European Atherosclerosis Society is highlighted by the specific Consensus by Gylling et al. [14]. This critical appraisal of the evidence about the benefit-to-risk relationship of functional foods with added plant sterols and/or plant stanols underlined that “daily consumption of foods with added plant sterols and/or plant stanols in amounts of up to 2 g/day is equally effective in lowering plasma atherogenic LDL-C levels by up to 10%, and thus may be considered as an adjunct to lifestyle in subjects at all levels of CV risk” [14] and that “plant sterols and plant stanols can be efficaciously combined with statins” [14], while “very limited data suggest plant sterols/stanols may also lower LDL-C levels in combination with a fibrate or ezetimibe” [14].

Another product that these guidelines proposed for the management of hypercholesterolemia is red yeast rice (RYR) extract, thanks to its bioactive ingredient, monacolin k, that shows a statin-like mechanism. Different commercial preparations of RYR, with various concentrations of monacolins, are available on the market. However, safety issues, due to the possible presence of contaminants in some preparations, have been raised. The guidelines state that “Nutraceuticals containing purified RYR may be considered in people with elevated plasma cholesterol concentrations who do not qualify for treatment with statins in view of their global CV risk” [4], but also warrant for better regulation of this kind of supplements.

The role of dietary fibres in cholesterol lowering has been strengthened in the last version of these guidelines (“Foods enriched with these fibres or supplements are well tolerated, effective, and recommended for LDL-C lowering” [4]) after the publication of the Cochrane meta-analysis in 2016 [15]. In contrast, the role of soy, suggested in the 2011 version of the ESC/EAS Guidelines as “substitute for animal protein foods high in SFAs” [16], even if “expected LDL-C lowering may be modest (3–5%) and most likely in subjects with hypercholesterolaemia” [16], was then downscored: “LDL-C-lowering effect […] was not confirmed when changes in other dietary components were taken into account” [4].

Finally, policosanol was described as showing “no significant effect on LDL-C, HDL-C, triglyceride (TG)” [4], and for berberine, despite a recent meta-analysis showing some lipid-lowering effect [17], “due to the lack of high-quality randomized clinical trials, its efficacy for treating dyslipidaemia needs to be further validated” [4].

Regarding the role of omega-3 polyunsaturated fatty acids (PUFA) in TG lowering, the recent guidelines reported that “Observational evidence indicates that consumption of fish (at least twice a week) and vegetable foods rich in omega-3 fatty acids […] is associated with lower risk of CV death and stroke, but has no major effects on plasma lipoprotein metabolism” [4] and that “Pharmacological doses of long-chain omega-3 fatty acids (2–3 g/day) reduce TG levels by about 30%” [4]. Concerning hard CV endpoints, the 2019 guidelines had the opportunity to integrate results from the REDUCE-IT trial [18] (where a significantly lower risk of ischaemic events was reported in patients with elevated TG levels treated with 4 g of icosapent ethyl), but not from the most recent RCTs on omega-3 unsaturated fatty acids [19,20].

The US Guidelines approach is quite different. In the 2018 AHA/ACC/AACVPR/AAPA/ABC/ACPM/ADA/AGS/APhA/ASPC/NLA/PCNA Guideline on the Management of Blood Cholesterol [21], there is no mention of nutraceuticals (whether general or specific products) and only a generic indication to pay attention to the diet (“Lifestyle counseling: use principles of Mediterranean and DASH diets” [22]). In 2018, the International Lipid Expert Panel (ILEP) published a Position Paper [23] defining the use of nutraceuticals in the management of statin intolerance: “Nutraceuticals, such as red yeast rice, bergamot, berberine, artichoke, soluble fiber, and plant sterols and stanols alone or in combination with each other, as well as with ezetimibe, might be considered as an alternative or add-on therapy to statins” [23]. Among the listed products, berberine, omega-3 PUFA and RYR were deemed as class of recommendation I, and therefore, enough supported by evidence of benefit and safety to be recommended/indicated. Nevertheless, also in this case, authors recognise the limitation of non-statin studies. In the Japan Atherosclerosis Society (JAS) Guidelines for Prevention of Atherosclerotic Cardiovascular Diseases 2017 [24], the section dedicated to lifestyle modification clearly stated that “increasing the intake of omega-3 PUFA is effective in decreasing the TG level and may lead to suppression of coronary artery disease (CAD)” [24], and that “Consuming soy and soy products is recommended because they may decrease CAD and stroke risk [24]”. Moreover, omega-3 PUFA are also indicated as treatment: “PUFAs are indicated for dyslipidaemia accompanied by an increased TG level, particularly for type Ⅱb and type Ⅳ hyperlipidemia” [24].

The recently released 2021 ESC Guidelines on cardiovascular disease prevention in clinical practice [25] also comment on the potential usefulness of nutraceutical product to reduce lipid levels: “An evidence-based approach to the use of lipid-lowering nutraceuticals could improve the quality of the treatment, including therapy adherence, and achievement of the LDL-C goal in clinical practice” [25]. However, importantly, they warn about the lack of outcome studies proving that nutraceuticals can prevent CVD morbidity or mortality [26].

In conclusion, some, but not all, guidelines for dyslipidaemia mention nutraceutical products as potential useful options for the treatment of mild dyslipidaemia, but also indicate the low level of evidence associated to their effects on hard endpoints, such as prevention of myocardial infarction and stroke.

**Table 1:** Nutraceutical products and guidelines for dyslipidaemia.

Nutraceutical Products Mentioned: Plant Sterols and Stanols, Red Yeast Rice Extract, Dietary Fibres, Soy, Policosanol, Berberine, Omega-3 Polyunsaturated Fatty Acids, Bergamot, Artichoke		
Year	Guideline (S) Name	Ref.
2019	ESC/EAS Guidelines for the management of dyslipidaemias	[4]
2018	AHA/ACC/AACVPR/AAPA/ABC/ACPM/ADA/AGS/APhA/ASPC/	[21]
	NLA/PCNA Guideline on the Management of Blood Cholesterol	
2018	International Lipid Expert Panel Position Paper	[23]
2017	Japan Atherosclerosis Society (JAS) Guidelines for Prevention of Atherosclerotic Cardiovascular Diseases	[24]
2011	ESC/EAS Guidelines for the management of dyslipidaemias	[16]

## 3. Nutraceutical Products and Guidelines for Obesity

The increasing prevalence of obesity and the related cardio-metabolic complications is well established. However, the practical prevention and management of this pathological condition is complex and difficult, due to the interplay of multiple and individually specific causative factors. This situation led to the development of a large series of approaches, ranging from pharmaceutical products to approaches with specific dietary programs or using plant extracts and food supplements (Table 2). The effectiveness and safety of these approaches still appear to be determined in many instances. In 2018, the Endocrine Society (USA) published a scientific statement on obesity management [27]. In this document, a detailed table listed a series of dietary supplements proposed for obesity management, defined in the text as “Dietary supplements, over-the-counter products, and other treatments with unproven efficacy and unknown safety” [27], which thus, have not undergone any FDA evaluation. As a word of caution, the authors suggested “Evidence to support the effectiveness for weight loss or the safety of these preparations is usually nonexistent. Moreover, variability in the composition of these products adds an additional uncertainty to their use. We thus think that the public would be better served if the dietary supplements were held to a higher standard and were overseen by the FDA” [27]. These considerations then indicate that, at least in the USA, no specific validation of such products has been conducted. The American Association of Clinical Endocrinologists (AACE) guidelines for the practical management of obesity, published in 2016 [28], do not report any food supplement or nutraceutical approach to obesity, but recommend that “Combinations of FDA-approved weight loss medications should only be used in a manner approved by the FDA or when sufficient safety and efficacy data are available to assure informed judgment regarding a favorable benefit-to-risk ratio” [28], again suggesting caution in such weight loss strategies.

The 2015 European Guidelines for Obesity Management in Adults [29], by the European Association for the Study of Obesity (EASO), mentions, in the section “Alternative therapies” that “[…] unorthodox and unproven treatments flourish and are often offered. There is insufficient evidence to recommend in favour of herbal medicines, dietary supplements or homoeopathy for obesity management in the obese person. Physicians should advise patients to follow evidence-based treatments and recommend treatments only where evidence of safety and efficacy has been established” [29]. As an additional strategy, subjects with obesity may benefit of very-low-calorie diet (VLCD) approaches [30], which, however, may be detrimental to some patients and, thus, should be conducted under the supervision of a physician. An EASO Position Statement published in 2014 [31] mentioned the VLCD approach, which has been recently evaluated in depth, with the first definition of the European Guidelines on this topic [32].

In conclusion, according to the most relevant and recent guidelines on obesity, no safe and effective dietary supplement nor nutraceutical product is available for the management of weight loss in this condition, and more high-quality studies are necessary in this field.

**Table 2:** Nutraceutical products and guidelines for obesity.

Nutraceutical Products Mentioned: “Dietary Supplements, Over-the-Counter Products, and Other Treatments” Ref. [31], Herbal Medicines, Dietary Supplements, Very-Low-Calorie Diet		
Year	Guideline (S) Name	Ref.
2018	Endocrine Society (USA) scientific statement on obesity management	[27]
2016	American Association of Clinical Endocrinologists guidelines for the practical management of obesity	[28]
2015	EASO European Guidelines for Obesity Management in Adults	[29]
2014	EASO Position Statement	[31]

## 4. Nutraceutical Products and Guidelines for Type 2 Diabetes Mellitus

The pharmacological management of T2DM has recently been expanded to several different classes of drugs (dipeptidyl peptidase-4 (DPP4) inhibitors, sodium-glucose cotransporter 2 (SGLT2) inhibitors, glucagon-like peptide-1 receptor agonists (GLP1-RA), etc.) in addition to metformin, sulfonilureas and alpha-glucosidase inhibitors [33]. Interestingly, some (mainly SGLT2 inhibitors and GLP-1 RA) in addition to optimal control of glucose metabolism, also offer a relevant degree of cardiovascular and renovascular protection [34]. Within this context, especially for subjects with pre-diabetes, insulin resistance and moderate metabolic syndrome, or as an add-on to drug therapy, several nutraceutical products have been proposed for glycaemic control (Table 3). These products include, for example, berberine [35], *Morus alba* extract [36] and other herbal extracts [37].

In 2021 and in 2022, the American Diabetes Association (ADA) released the updated “Standards of Medical Care in Diabetes”. The 2021 and 2022 sections devoted to pharmacologic approaches [33,38] report in great detail the pharmacological options for the management of T2DM, but do not mention the use of any supplement or nutraceutical product, either in a positive or negative way. In the section of these “Standards of Medical Care in Diabetes” devoted to cardiovascular disease and risk management [39,40], the authors include, in the 10.15 Recommendation, some dietary suggestions, including “[…] increase of dietary omega-3 fatty acids, viscous fiber, and plant stanols/sterols intake […]” [40], thus generically mentioning some nutraceuticals discussed in greater detail in the guidelines devoted to the management of dyslipidaemia (see above).

In the “2019 ESC Guidelines on diabetes, pre-diabetes, and cardiovascular diseases developed in collaboration with the European Association for the Study of Diabetes (EASD)” [34], a specific reference is present indicating that “Supplements with omega-3 fatty acids have not been shown to improve glycaemic control in individuals with DM and RCTs do not support recommending omega-3 supplements for the primary or secondary prevention of CVD” [34]. Moreover, a specific comment on the positive outcome of the REDUCE-IT trial [18], discussed above, on the primary endpoint of major adverse CV events (MACE) is also included. In 2018, a consensus report by the ADA and the EASD on “Management of hyperglycaemia in type 2 diabetes” was published [41]. This very detailed document does not mention, however, any food supplement or nutraceutical product proposed or even not recommended for T2DM management. Similarly, no reference to nutraceuticals or food supplements is present in the 2017 “Recommendations for Managing Type 2 Diabetes in Primary Care” by the International Diabetes Federation [42].

Obesity and T2DM may often be associated with non-alcoholic fatty liver disease (NAFLD). On this topic, in 2016, the European Association for the Study of the Liver (EASL), the EASD and the The European Association for the Study of Obesity (EASO) published the Clinical Practice Guidelines [43], which did not mention any nutraceutical product/food supplement.

In conclusion, guidelines for T2DM do not mention any specific nutraceutical approach to this disease, nor to milder forms such as insulin resistance and pre-diabetes, which may be observed in the early phases before proper T2DM development. In any case, if the validation of such products is considered important, robust clinical research will, thus, need to be implemented in this specific area.

**Table 3:** Nutraceutical products and guidelines for type 2 diabetes mellitus.

Nutraceutical Products Mentioned: Berberine, Morus Alba Extract, other Herbal Extracts, Omega-3 Polyunsaturated Fatty Acids, Viscous Fibre, Plant Stanols/Sterols		
Year	Guideline (S) Name	Ref.
2021–2022	American Diabetes Association “Standards of Medical Care in Diabetes”	
	Pharmacologic Approaches to Glycemic Treatment	[33,38]
	Cardiovascular Disease and Risk Management	[39,40]
2019	ESC Guidelines on diabetes, pre-diabetes and cardiovascular diseases developed in collaboration with the EASD	[34]
2018	Management of hyperglycaemia in type 2 diabetes	[41]
	A consensus report by ADA and EASD	
2017	IDF Recommendations for Managing Type 2 Diabetes in Primary Care	[42]
2016	EASL–EASD–EASO Clinical Practice Guidelines for the management of non-alcoholic fatty liver disease	[43]

## 5. Concluding Remarks

In recent years, the use of nutraceuticals has become increasingly substantial in several areas of medicine [44]. In parallel, the prescription of these products by physicians has also increased [45]. However, the focus on nutraceuticals and dietary supplements in the guidelines dedicated to disease management remains limited, both in general and, more specifically, regarding cardio-metabolic diseases and the related features, such as dyslipidaemia. The preparation and implementation of guidelines represent a pivotal aspect of the translation of research-derived knowledge and evidence-based medicine to the clinical practice. This process requires a set of subsequent steps that need to be accurately defined and implemented [46,47,48], starting from robust evidence that is the background of useful and effective guidelines. As indicated by the GRADE system, there are four levels of evidence. Evidence from RCTs is of the highest quality and, because of residual confounding, evidence that includes observational data is considered to be low quality. However, the literature has consistently claimed that there are few RCT data considering hard end-points to establish clinical benefits [49]. This may be partly due to the current methodological shortcomings of RCT study design and the complex characteristics of nutraceuticals. In contrast to pharmaceutical trials, clinical trials on nutraceuticals and on enriched dietary patterns show critical challenges in terms of study design and methodology, tiny effect sizes, high heterogeneity of the responses and limited translatability of the observed effect size. These observations prompt further studies, leading to an improvement of the methodology, aiming at building a stronger evidence-based data that supports the use of these products in cardiovascular and metabolic prevention and, possibly, management.

## Data Availability

Not applicable.
